# Interocular Differences in Visual Acuity and Refractive Status Between Unilateral Early and Late Age-Related Macular Degeneration: A Nationwide Population-Based Study

**DOI:** 10.3390/jcm15197672

**Published:** 2026-10-03

**Authors:** Chang Gu Kang, Su Kang Kim, Hyungoo Kang, Sang-il Park

**Affiliations:** 1 Department of Biomedical Laboratory Science, Catholic Kwandong University, Gangneung 25601, Republic of Korea; 2 Institute of Natural Product Biotechnology, Catholic Kwandong University, Gangneung 25601, Republic of Korea; 3 Department of Optometry, Catholic Kwandong University, Gangneung 25601, Republic of Korea

**Keywords:** age-related macular degeneration, interocular visual acuity difference, refractive status, anisometropia, Korea national health and nutrition examination survey

## Abstract

**Background/Objectives:** Age-related macular degeneration (AMD) may affect the two eyes asymmetrically, but visual impairment is commonly classified by visual acuity (VA) in the better-seeing eye. We compared interocular-presenting VA differences in unilateral early versus late AMD, assessed the association between late AMD and worse VA in the AMD-affected eye, and compared refractive status between the affected and fellow eyes. **Methods:** We analyzed 1112 adults aged ≥40 years with unilateral AMD (1049 early and 63 late) from the 2017–2020 Korea National Health and Nutrition Examination Survey. Interocular VA difference was calculated as the logMAR VA of the AMD-affected eye minus that of the fellow eye. Survey-weighted modified Poisson regression estimated prevalence ratios (PRs), adjusting for age, sex, anisometropia, and the interocular difference in absolute spherical equivalent refraction. Exploratory analyses examined neovascular AMD and geographic atrophy separately, and sensitivity analyses assessed alternative model specifications. **Results:** Mean interocular differences were −0.004 logMAR (95% confidence interval [CI], −0.016 to 0.008) in early AMD and 0.184 logMAR (95% CI, 0.077 to 0.291) in late AMD. The weighted proportions with VA ≥ 0.2 logMAR worse in the AMD-affected eye were 10.15% and 58.31%, respectively. The adjusted PR for this outcome was 4.95 (95% CI, 3.46–7.09; *p* < 0.001) for late versus early AMD. Exploratory subtype-specific analyses showed similar associations for neovascular AMD and geographic atrophy. No statistically significant interocular differences in refractive status or astigmatism severity were detected; however, the wide 95% CIs, particularly in the late AMD group, precluded conclusions of equivalence. **Conclusions**: Unilateral late AMD was associated with greater interocular presenting VA asymmetry than early AMD, and the association remained consistent across sensitivity analyses. However, these cross-sectional findings do not establish longitudinal progression or directly demonstrate implications for spectacle prescribing or binocular visual function.

## 1. Introduction

Age-related macular degeneration (AMD) is a degenerative disease affecting the photoreceptors, retinal pigment epithelium (RPE), Bruch’s membrane, and choriocapillaris in the macula. It is a leading cause of irreversible vision loss in older adults [1]. Early AMD is characterized by drusen and RPE abnormalities, whereas late AMD involves macular neovascularization or geographic atrophy and can cause severe central vision loss [1]. The global number of individuals with AMD was projected to increase from approximately 196 million in 2020 to 288 million by 2040 [2]. AMD is among the leading causes of blindness and visual impairment worldwide, and its burden is expected to increase as the population ages [3].

AMD can affect both eyes, but it does not necessarily develop at the same time or progress to the same stage in each eye. An analysis of 13,737 Korean adults aged ≥40 years from KNHANES described the prevalence and patterns of bilateral AMD, including discordant stages between eyes, but did not examine functional differences between the AMD-affected eye and the fellow eye [4]. Visual impairment, however, is often classified according to VA in the better-seeing eye. This eye partly compensates for reduced vision in the other during daily activities, yet unilateral visual impairment still affects reading, facial recognition, and independence [5]. Impairment in the worse-seeing eye also affects vision-related quality of life, so assessment based on one eye alone underestimates the impact of visual loss [6]. In macular disease, interocular differences in visual function can reduce binocular summation or lead to binocular inhibition [7,8]. Both VA in each eye and the interocular difference therefore warrant assessment. When evaluating interocular VA differences in individuals with AMD, refractive status in both eyes should also be considered. Refractive errors, including myopia, hyperopia, and astigmatism, can influence measured logMAR VA, while anisometropia may contribute to interocular VA differences independently of AMD. In older adults, age-related changes in the crystalline lens and the development of cataracts can alter spherical and cylindrical refractive power. Asymmetric changes between the eyes increase the degree of anisometropia [9,10]. A longitudinal study of older adults reported that interocular differences in spherical equivalent refraction and cylindrical power increased with age [9]. These factors are therefore relevant when evaluating visual impairment in the AMD-affected eye. In addition to influencing VA measurements, refractive status has also been reported to be associated with AMD [11,12]. In a meta-analysis by Pan et al., hyperopia was associated with higher odds of prevalent AMD and myopia with lower odds in cross-sectional studies, although neither association reached significance in longitudinal cohorts [11]. Similarly, a study by Lin et al. using data from the KNHANES found that myopia was inversely associated with early AMD but was not significantly associated with late AMD [12]. These studies primarily examined population-level associations between refractive status and AMD, with relatively little attention given to interocular relationships in individuals with unilateral AMD. When refractive status differs between the eyes, the observed interocular VA difference may reflect both AMD-related impairment and refractive asymmetry, making it difficult to attribute the difference to AMD severity alone. Direct interocular comparison of VA between the AMD-affected eye and the fellow eye, together with assessment of interocular refractive differences, is thus needed.

Using nationally representative data from the 2017–2020 KNHANES, this study aimed to compare interocular VA differences between individuals with unilateral early AMD and those with unilateral late AMD. We then examined whether late AMD was associated with worse VA in the AMD-affected eye after adjustment for anisometropia and interocular refractive differences. Because late AMD comprises clinically distinct phenotypes, we additionally conducted exploratory analyses of neovascular AMD and geographic atrophy separately. In addition, we compared the proportions of myopia, emmetropia, and hyperopia and the proportions within each astigmatism category between the AMD-affected eye and the fellow eye. These comparisons may help identify interocular VA deficits that are not apparent from assessment of the better-seeing eye alone and provide a basis for further studies of binocular visual function in unilateral AMD.

## 2. Materials and Methods

### 2.1. Source Population

This cross-sectional study used data from the 2017–2020 KNHANES, conducted by the Korea Disease Control and Prevention Agency. KNHANES is an annual nationwide survey designed to assess the health and nutritional status of the Korean population. Participants are selected using a stratified, multistage cluster probability sampling design [13,14]. Individuals aged ≥40 years with available data on AMD status, VA, and refraction in both eyes, as well as age and sex, were initially identified. Among them, individuals with unilateral AMD, defined as AMD in one eye and no AMD in the fellow eye, were included in the final analysis. Individuals with bilateral AMD, no AMD in either eye, ungradable AMD status, or missing data required for the analysis were excluded.

KNHANES was conducted in accordance with relevant ethical standards, and written informed consent was obtained from all participants. The present study was granted an exemption from review by the Institutional Review Board of Catholic Kwandong University (IRB No. CKU-25-02-1002). Publicly available, de-identified data were used for the analysis.

### 2.2. AMD Assessment and Classification

AMD status was determined using the ophthalmic examination data provided by KNHANES. Across the 2017–2020 survey cycles, ophthalmic examinations, including VA assessment, autorefraction, and fundus imaging, were conducted according to the standardized KNHANES protocols described for KNHANES VII and VIII [15]. AMD classification was based primarily on nonmydriatic fundus photographs, with optical coherence tomography (OCT) images used as supplementary information, and the images were graded by ophthalmologists [15]. Early AMD was defined by the presence of soft indistinct or reticular drusen, or hard or soft distinct drusen accompanied by RPE pigmentary abnormalities, including increased pigmentation or hypopigmentation, in the absence of signs of late AMD [15]. Late AMD included geographic atrophy (late dry AMD) and neovascular AMD (wet AMD) [16]. Geographic atrophy and neovascular AMD were combined in the primary late-AMD category in accordance with the conventional classification of late AMD and to preserve statistical precision given the small subtype-specific sample sizes; the two subtypes were additionally examined separately in exploratory analyses. Among individuals with unilateral AMD, those whose AMD-affected eye was classified as having early AMD were assigned to the early AMD group, whereas those with geographic atrophy or neovascular AMD were assigned to the late AMD group. The fellow eye was required to be free of AMD. Individuals with bilateral AMD or unassessable AMD status in either eye were excluded. For the sensitivity analysis, participants with a recorded positive classification for diabetic retinopathy, diabetic macular edema, epiretinal membrane, macular hole, or retinal vein occlusion in either eye were excluded based on the KNHANES retinal grading variables [15].

### 2.3. Visual Acuity and Interocular Difference

Presenting distance VA data were obtained from KNHANES. VA was measured separately in each eye at 4 m using a Snellen-based chart, either without correction or with the participant’s habitual spectacles or contact lenses [15]. Presenting VA was used because consistently measured best-corrected VA was not available in KNHANES; therefore, the measured VA could reflect residual uncorrected or undercorrected refractive error in addition to retinal dysfunction. Decimal VA values were converted to logMAR units using the formula logMAR = −log10 (decimal VA). The interocular VA difference was calculated as the logMAR VA of the AMD-affected eye minus that of the fellow eye. A positive value indicated worse VA in the AMD-affected eye, whereas a negative value indicated better VA in the AMD-affected eye. Mean interocular differences were calculated only for participants with quantifiable VA in both eyes. When VA in the AMD-affected eye was off-chart (counting fingers, hand motion, light perception, or no light perception) but VA in the fellow eye was quantifiable, the participant was classified as meeting all three VA difference thresholds. In the reverse situation, the participant was classified as not meeting any of the thresholds. No participant had off-chart VA in both eyes. Because off-chart categories were recorded categorically rather than measured on a common logMAR scale, numeric logMAR values were not assigned. Instead, directional threshold classification was used to avoid imposing arbitrary numeric values, and its influence was evaluated in sensitivity analyses restricted to participants with quantifiable VA in both eyes. The proportions of participants whose AMD-affected eye had VA ≥0.1, ≥0.2, or ≥0.3 logMAR worse than the fellow eye were calculated. The ≥0.2 logMAR threshold, corresponding to approximately two lines on a logMAR chart, was selected a priori as the primary outcome. In repeated ETDRS logMAR testing of the same eye, differences in this magnitude have been reported to be distinguishable from test–retest variability [17]. Although this evidence derives from intra-eye repeatability rather than interocular comparison, a two-line threshold was considered a conservative criterion for identifying an interocular difference beyond measurement variability, particularly because VA in KNHANES was measured using a Snellen-based chart rather than ETDRS charts. The ≥0.1 and ≥0.3 logMAR thresholds were examined in sensitivity analyses to assess the robustness of the findings to the outcome definition.

### 2.4. Refraction and Classification of Refractive Status

Spherical and cylindrical powers measured by automated refractometry were used in the analysis [15]. To account for differences in cylinder sign conventions across survey years, cylindrical power was standardized to minus-cylinder form before calculating spherical equivalent refraction. Spherical equivalent refraction (SER), calculated as sphere plus one-half of the signed cylinder power, was used to classify refractive status. Myopia was defined as an SER of ≤−0.50 D, emmetropia as an SER of >−0.50 D and <0.50 D, and hyperopia as an SER of ≥0.50 D. Astigmatism was classified according to the absolute cylindrical power as <0.50 D, ≥0.50 to <1.00 D, ≥1.00 to <2.00 D, or ≥2.00 D. Anisometropia was defined as an absolute interocular difference in SER of ≥1.00 D.

### 2.5. Adjustment Variables

Covariates considered in the analyses included age, sex, anisometropia, the interocular difference in absolute SER, physician-diagnosed cataract, and history of cataract surgery. Age was treated as a continuous variable, and sex was categorized as male or female. The interocular refractive difference was calculated by subtracting the absolute SER of the fellow eye from that of the AMD-affected eye. For example, if the AMD-affected eye had an SER of −3.00 D and the fellow eye an SER of +1.00 D, the interocular refractive difference was +2.00 D (3.00–1.00), whereas the absolute interocular SER difference used to define anisometropia was 4.00 D. Conversely, SERs of +2.00 D and −2.00 D correspond to 4.00 D of anisometropia but an interocular refractive difference of 0.00 D. Thus, anisometropia reflects the overall magnitude of refractive asymmetry regardless of which eye had the larger refractive error, whereas the interocular refractive difference indicates whether the refractive error was larger in magnitude in the AMD-affected eye, which could itself contribute to worse presenting VA in that eye. Both variables were therefore included in the adjusted models. Physician-diagnosed cataract was determined from participants’ responses to whether they had ever been diagnosed with cataract by a physician, and history of cataract surgery was determined from responses to the questionnaire on ocular surgery [15]. VA testing condition was classified as uncorrected or measured with habitual spectacles or contact lenses. In the sensitivity analysis, participants with different or unavailable VA testing conditions between eyes were excluded, and habitual optical correction was included as a binary covariate. Participants with missing cataract-related information were excluded from analyses that included the corresponding cataract-related covariates.

### 2.6. Statistical Analysis

All analyses accounted for the stratification, clustering, and sampling weights of KNHANES [15]. Sample sizes were reported as unweighted counts, whereas continuous and categorical variables were summarized as survey-weighted means and proportions with standard errors, respectively. Differences in mean interocular VA and weighted proportions between the early and late AMD groups were evaluated using design-based Wald tests. Survey-weighted modified Poisson regression with a log link and design-based robust variance estimation was used to calculate prevalence ratios (PRs) and 95% confidence intervals (CIs) [18]. Prevalence ratios were preferred to odds ratios because the outcome was not rare and odds ratios could overstate the magnitude of the cross-sectional association. Models were sequentially adjusted for age and sex, anisometropia, and the interocular refractive difference. Cataract diagnosis and history of cataract surgery were additionally included in a separate model. The ≥0.2 logMAR threshold was used for the primary analysis, whereas the ≥0.1 and ≥0.3 logMAR thresholds were examined in sensitivity analyses. To evaluate the influence of off-chart VA classification, additional sensitivity analyses restricted to participants with quantifiable VA in both eyes were performed using Model 3 at the ≥0.1, ≥0.2, and ≥0.3 logMAR thresholds. In an additional sensitivity analysis, participants with different or unavailable VA testing conditions between eyes were excluded, and habitual optical correction during VA testing was included as an adjustment variable. Multicollinearity among the covariates in Model 3 was assessed using variance inflation factors (VIFs), with values < 5 considered acceptable. To assess the robustness of the adjustment for refractive asymmetry, alternative models included both anisometropia and the continuous interocular difference in absolute SER, either refractive covariate alone, or neither refractive covariate. In an additional model, dichotomous anisometropia was replaced with the continuous absolute interocular difference in SER, while the interocular difference in absolute SER was retained. A further sensitivity analysis additionally adjusted Model 3 for survey year as a categorical variable. Because late AMD comprises clinically distinct phenotypes, exploratory subtype-specific analyses compared neovascular AMD and geographic atrophy separately with early AMD using Model 3. Kish effective sample sizes, calculated as the squared sum of the survey weights divided by the sum of their squared values, were used to assess precision under unequal weighting. Participants included in the complete-case analysis were also descriptively compared with those excluded because of missing data in terms of age, sex, and the prevalence of any AMD. No formal statistical testing was performed for this descriptive comparison. Paired interocular differences in refractive status proportions were calculated as the AMD-affected eye minus the fellow eye and are presented as survey-weighted percentage-point differences with 95% CIs. The refractive-status analyses were exploratory, and no adjustment for multiple comparisons was performed. Accordingly, interpretation emphasized effect estimates and 95% confidence intervals rather than isolated *p* values. A sensitivity analysis excluding participants with retinal diseases other than AMD was also performed using Model 3. Statistical analyses were performed using IBM SPSS Statistics version 29.0 (IBM Corp., Armonk, NY, USA) and Python version 3.12 (Python Software Foundation, Wilmington, DE, USA). All statistical tests were two-sided, and *p* < 0.05 was considered statistically significant.

## 3. Results

### 3.1. Participant Selection

**Figure 1:**
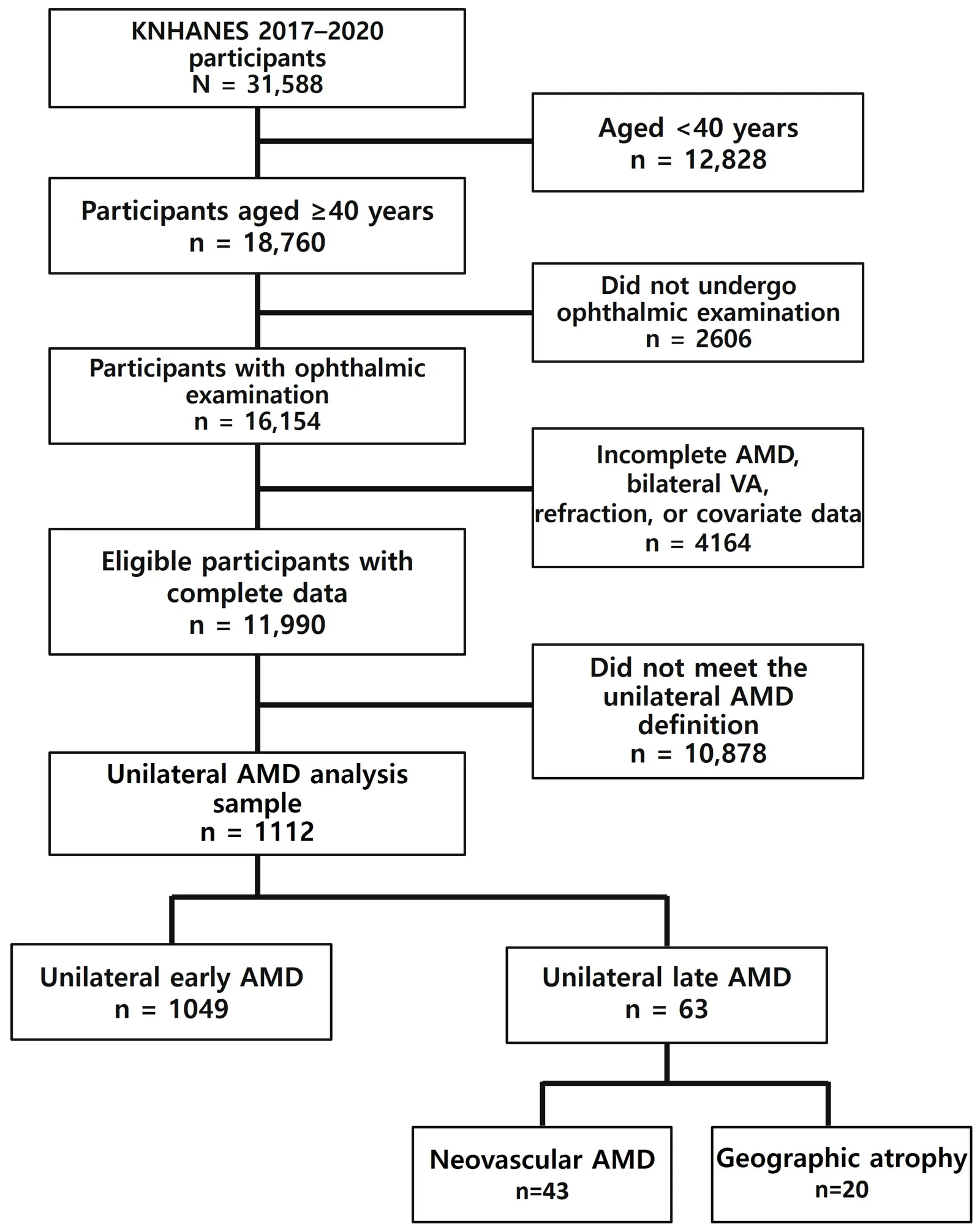
Flow diagram of participant selection from the Korea National Health and Nutrition Examination Survey, 2017–2020. AMD, age-related macular degeneration; KNHANES, Korea National Health and Nutrition Examination Survey; VA, visual acuity.

A total of 31,588 individuals participated in the 2017–2020 KNHANES. After excluding 12,828 individuals aged <40 years and 2606 who did not undergo ophthalmic examinations, 16,154 remained. Further restriction to individuals with complete data required for the analysis, including AMD status, VA, and refraction in both eyes, resulted in 11,990 eligible participants. Among the 4164 participants excluded because of incomplete data, AMD status was unavailable for 1062, bilateral VA data were unavailable for 192, and bilateral refraction data were unavailable for 3436; these categories were not mutually exclusive. Age and sex data were complete (Table S1). Of these, 1112 participants with AMD in one eye and no AMD in the fellow eye were included in the final analysis. The final study population comprised 1049 participants with early AMD and 63 with late AMD. Among those with late AMD, 43 had neovascular AMD and 20 had geographic atrophy (Figure 1). Compared to the 4164 participants excluded because of missing required data, the 11,990 participants included in the complete-case analysis were slightly older (survey-weighted mean age, 57.89 vs. 56.52 years) and more frequently female (52.51% vs. 49.93%). AMD status was ascertainable for 3102 of the excluded participants. The survey-weighted prevalence of any AMD among participants with ascertainable AMD status was 15.24% in the complete-case group and 11.29% in the excluded group (Table S1).

### 3.2. Participant Characteristics

The survey-weighted mean age was 63.53 ± 0.41 years in the early AMD group and 68.00 ± 1.55 years in the late AMD group. The weighted proportions of female participants were 44.81 ± 1.74% and 27.10 ± 6.11%, respectively. The weighted proportions of participants with anisometropia of ≥1.0 D were 15.66 ± 1.27% in the early AMD group and 34.90 ± 6.74% in the late AMD group. The survey-weighted mean difference between the absolute spherical equivalent refraction values of the AMD-affected and fellow eyes, calculated as the affected eye minus the fellow eye, was −0.066 ± 0.043 D in the early AMD group and −0.123 ± 0.202 D in the late AMD group. The weighted proportions of participants with cataract diagnosis were 25.86 ± 1.61% and 42.60 ± 7.23%, respectively, while the corresponding proportions with a history of cataract surgery were 4.59 ± 0.77% and 9.62 ± 4.60%. Data on cataract diagnosis were available for 1023 participants with early AMD and 61 with late AMD and were missing for 26 and two participants, respectively (Table 1).

### 3.3. Interocular Visual Acuity Differences in Early and Late AMD

VA could be quantified in logMAR units for both eyes in 1035 participants with early AMD and 48 with late AMD. Among the remaining 29 participants, 16 had off-chart VA only in the AMD-affected eye (one with early AMD and 15 with late AMD), whereas 13 had off-chart VA only in the fellow eye (all with early AMD). No participant had off-chart VA in both eyes. The survey-weighted mean interocular VA difference, calculated as the logMAR VA of the AMD-affected eye minus that of the fellow eye, was −0.004 logMAR (95% CI, −0.016 to 0.008) in the early AMD group and 0.184 logMAR (95% CI, 0.077 to 0.291) in the late AMD group. The weighted proportion of participants whose VA in the AMD-affected eye was ≥0.1 logMAR worse than that in the fellow eye was 17.12% (95% CI, 14.46 to 19.77) in the early AMD group and 61.85% (95% CI, 48.29 to 75.42) in the late AMD group. At the ≥0.2 logMAR threshold, the corresponding proportions were 10.15% (95% CI, 8.17 to 12.14) and 58.31% (95% CI, 44.32 to 72.29), respectively. At the ≥0.3 logMAR threshold, they were 4.54% (95% CI, 3.18 to 5.90) and 46.28% (95% CI, 31.93 to 60.63), respectively. The mean interocular VA difference and the weighted proportions at all three thresholds differed significantly between the early and late AMD groups (all *p* < 0.001) (Table 2).

### 3.4. Association Between Late AMD and Worse Visual Acuity in the AMD-Affected Eye

In the age- and sex-adjusted model, the PR for VA ≥0.2 logMAR worse in the AMD-affected eye was 5.31 for late AMD compared with early AMD (95% CI, 3.72 to 7.60; *p* < 0.001). After additional adjustment for anisometropia, the PR was 5.02 (95% CI, 3.49 to 7.21; *p* < 0.001). After further adjustment for the interocular refractive difference, the PR was 4.95 (95% CI, 3.46 to 7.09; *p* < 0.001). When the analysis was restricted to 1083 participants with quantifiable VA in both eyes, the PRs at the ≥0.1, ≥0.2, and ≥0.3 logMAR thresholds were 2.93 (95% CI, 2.06 to 4.17), 4.49 (95% CI, 2.94 to 6.87), and 6.29 (95% CI, 3.42 to 11.56), respectively (all *p* < 0.001) (Table 3). In a separate analysis of 1084 participants with available cataract-related information, additional adjustment for cataract diagnosis and history of cataract surgery yielded a PR of 5.29 (95% CI, 3.72 to 7.53; *p* < 0.001). At the ≥0.1, ≥0.2, and ≥0.3 logMAR thresholds, the PRs comparing late AMD with early AMD were 3.26 (95% CI, 2.42 to 4.37; *p* < 0.001), 4.95 (95% CI, 3.46 to 7.09; *p* < 0.001), and 7.77 (95% CI, 4.78 to 12.63; *p* < 0.001), respectively. After excluding 206 participants with recorded retinal diseases other than AMD (190 with early AMD and 16 with late AMD), the results remained similar among the remaining 906 participants (PR, 5.20; 95% CI, 3.28 to 8.23; *p* < 0.001). A separate sensitivity analysis restricted to 1109 participants with the same VA testing condition in both eyes and adjusted for habitual optical correction during testing yielded a similar result (PR, 5.01; 95% CI, 3.48 to 7.21; *p* < 0.001) (Table 3). The Kish effective sample sizes were 758.55 for early AMD and 46.51 for late AMD overall; the corresponding values were 30.62 for neovascular AMD and 16.26 for geographic atrophy. In the subtype-specific descriptive analyses, the mean interocular differences were 0.188 logMAR (95% CI, 0.047 to 0.330) for neovascular AMD and 0.175 logMAR (95% CI, 0.028 to 0.323) for geographic atrophy. The corresponding weighted proportions meeting the ≥0.2 logMAR threshold were 57.04% (95% CI, 39.37% to 74.70%) and 60.30% (95% CI, 36.20% to 84.40%), respectively; off-chart VA in the AMD-affected eye was observed in 9 and 6 participants, respectively (Table S2). In exploratory subtype-specific analyses, the adjusted PRs were 4.84 for neovascular AMD versus early AMD (95% CI, 3.21 to 7.29; *p* < 0.001) and 5.13 for geographic atrophy versus early AMD (95% CI, 3.12 to 8.44; *p* < 0.001) (Table S2). All VIFs in Model 3 ranged from 1.01 to 1.06, indicating no meaningful multicollinearity among the covariates, including anisometropia and the interocular difference in absolute SER. The association was also consistent across alternative specifications of the refractive covariates, including replacement of dichotomous anisometropia with the continuous absolute interocular difference in SER, with adjusted PRs ranging from 4.95 to 5.37 (all *p* < 0.001). Additional adjustment of Model 3 for survey year yielded a similar estimate (PR, 5.42; 95% CI, 3.71 to 7.91; *p* < 0.001) (Table S3).

### 3.5. Interocular Comparison of Refractive Status Categories

Refractive status in each eye was classified as myopia, emmetropia, or hyperopia based on spherical equivalent refraction (SER). In the early AMD group, the proportion classified as myopic was 1.96 percentage points higher in the AMD-affected eye (95% CI, −1.02 to 4.95). The proportions classified as emmetropic and hyperopic were lower in the AMD-affected eye by 1.45 percentage points (95% CI, −4.95 to 2.04) and 0.51 percentage points (95% CI, −2.94 to 1.93), respectively. In the late AMD group, the proportion classified as myopic was 0.52 percentage points higher in the AMD-affected eye (95% CI, −14.85 to 15.89), whereas the proportion classified as emmetropic was 2.60 percentage points lower (95% CI, −18.61 to 13.40). The proportion classified as hyperopic was 2.08 percentage points higher in the AMD-affected eye (95% CI, −8.36 to 12.53). Interocular differences in the proportions across astigmatism severity categories ranged from −0.67 to 0.79 percentage points in the early AMD group and from −8.08 to 7.17 percentage points in the late AMD group. All 95% CIs for these interocular proportion differences included zero, and no statistically significant interocular differences were observed in refractive status or astigmatism severity categories in either group. However, the estimates for the late AMD group were imprecise, as indicated by the wide 95% CIs (Table 4). These analyses were exploratory and were not adjusted for multiple comparisons; therefore, the nonsignificant results should not be interpreted as evidence of equivalence or as excluding clinically relevant interocular differences.

## 4. Discussion

The mean interocular VA difference was minimal in the early AMD group, yet 17.12% of these participants had VA at least 0.1 logMAR worse in the AMD-affected eye. In the late AMD group, both the mean interocular VA difference and the proportions of participants with VA ≥0.1, ≥0.2, and ≥0.3 logMAR worse in the AMD-affected eye were markedly greater than those in the early AMD group. The mean difference of 0.184 logMAR corresponds to approximately two 0.1-logMAR lines. However, this approximate line-based interpretation should be viewed cautiously because VA was measured using a Snellen-based chart under presenting conditions. In the late AMD group, 46.28% met the ≥0.3 logMAR criterion, which also included participants with off-chart VA in the AMD-affected eye and quantifiable VA in the fellow eye. The adjusted PR for the ≥0.2 logMAR threshold was 4.95 for late AMD compared with early AMD. In contrast, no statistically significant interocular differences were observed in the proportions classified as myopic, emmetropic, or hyperopic or across astigmatism severity categories, although the estimates for the late AMD group were imprecise because of the small sample size. Exploratory subtype-specific analyses showed similarly increased prevalence ratios for neovascular AMD and geographic atrophy compared with early AMD, suggesting that the overall late-AMD association was not attributable to only one subtype. However, the small numbers and low effective sample sizes of both late-AMD subtypes preclude definitive comparisons between neovascular AMD and geographic atrophy.

In early AMD, low-luminance VA, dark adaptation, and color vision can be impaired even when conventional high-contrast VA is preserved [19]. In macular disease, the better-seeing eye tends to dominate binocular function as the functional difference between the eyes widens, although some patients instead show reduced binocular summation or binocular inhibition [7]. Binocular summation and inhibition in AMD vary considerably between individuals, and interocular VA differences contribute to this variation [20]. Binocular summation and binocular inhibition were not directly measured in the present study and are discussed only as potential clinical implications. Among patients with central vision loss, those exhibiting binocular inhibition had greater interocular VA differences and slower reading speeds under binocular viewing conditions [8]. Therefore, assessment of the fellow eye alone may not fully describe visual impairment in individuals with unilateral AMD. The association between late AMD and worse VA in the AMD-affected eye remained after adjustment for anisometropia and the interocular refractive difference. Similar estimates were obtained in separate analyses involving additional adjustment for cataract diagnosis and history of cataract surgery, exclusion of participants with other retinal diseases, restriction to participants with quantifiable VA in both eyes, and accounting for the VA testing condition. The estimates also remained consistent across alternative specifications of the refractive covariates and after additional adjustment for survey year. When refractive correction alone is insufficient, visual rehabilitation and optical aids offer a further option for patients with central vision loss [21]. A systematic review reported improvements in VA, reading performance, and facial recognition with low vision devices in patients with AMD [22]. The survey-weighted proportion of participants with anisometropia was higher in the late AMD group than in the early AMD group (34.90% vs. 15.66%). The cross-sectional design does not establish the reason for this difference. Although spectacle tolerance and binocular function were not directly evaluated, interocular VA differences and anisometropia may warrant consideration when planning refractive correction for patients with unilateral late AMD. Prospective studies directly assessing binocular visual function, subjective visual discomfort, and adaptation to spectacle correction are needed before specific prescribing recommendations can be made.

This study compared the AMD-affected and fellow eyes within the same participants using nationally representative data, which limits confounding by between-person characteristics. Several limitations remain. The cross-sectional design precludes establishing temporal or causal relationships and does not permit inference regarding visual decline or disease progression. The marked imbalance between the early- and late-AMD groups (1049 vs. 63 participants), together with a Kish effective sample size of 46.51 for the combined late-AMD group, limited the precision of the estimates, as reflected in the wide confidence intervals. Although the effect estimates remained consistent across sensitivity analyses, findings for late AMD, particularly the subtype-specific estimates, should be interpreted cautiously. The complete-case approach may also have introduced selection bias. Compared to participants excluded because of missing data, those included in the complete-case analysis were slightly older, more frequently female, and had a higher prevalence of any AMD among participants with ascertainable AMD status. Although these differences were modest, residual differences between the included and excluded participants cannot be excluded. The mean interocular VA difference could be calculated only for participants with VA quantifiable in logMAR units in both eyes, and those with nonquantifiable VA were therefore excluded from this calculation. VA was also assessed under presenting rather than best-corrected conditions, leaving residual effects of uncorrected or undercorrected refractive error possible. However, 1109 of 1112 participants (99.7%) had the same VA testing condition in both eyes, and additional adjustment for habitual optical correction did not materially alter the results. The present analysis was also limited to high-contrast chart VA and did not evaluate other aspects of visual function, such as contrast sensitivity, low-luminance VA, and dark adaptation. Although cataract history was included in the adjusted analysis, eye-specific cataract status and other coexisting ocular conditions could not be fully accounted for. The findings may not be generalizable to patients with bilateral AMD. In addition, information on lesion location and extent, neovascular activity, and anti-vascular endothelial growth factor treatment was unavailable, and residual confounding by unmeasured eye-specific ocular conditions cannot be excluded. Future longitudinal studies should include a larger number of participants with late AMD and evaluate additional aspects of visual function to characterize changes in interocular visual function as AMD progresses. Statistical adjustment for refractive variables cannot reproduce the clinical information provided by consistently measured best-corrected VA. Cataract information was not eye-specific and did not include lens-opacity severity; therefore, residual confounding due to asymmetric media opacity cannot be excluded. Other eye-specific ocular conditions were not comprehensively available in KNHANES and could not be controlled for.

## 5. Conclusions

Using nationally representative data from the 2017–2020 KNHANES, this study compared VA and refractive status between the AMD-affected and fellow eyes in individuals with unilateral AMD. Participants with late AMD had more pronounced interocular VA differences than those with early AMD, and the association between late AMD and worse VA in the AMD-affected eye persisted after adjustment for age, sex, anisometropia, and interocular refractive differences. No statistically significant interocular differences in refractive-category proportions were detected; however, the wide confidence intervals, particularly in the late-AMD group, precluded conclusions of equivalence or the exclusion of clinically relevant differences. These cross-sectional findings characterize stage-related interocular VA asymmetry but do not establish longitudinal progression or directly demonstrate implications for spectacle prescribing or binocular visual function. Prospective studies incorporating best-corrected VA and direct measures of binocular function are warranted. A small interocular difference in high-contrast chart VA should not be interpreted as indicating minimal asymmetry in overall visual function.

## Figures and Tables

**Table 1:** Characteristics of participants with unilateral early and late age-related macular degeneration.

Characteristic	Early AMD (*n* = 1049)	Late AMD (*n* = 63)
Age (y)	63.53 ± 0.41	68.00 ± 1.55
Female (%)	44.81 ± 1.74	27.10 ± 6.11
Anisometropia ≥ 1.0 D (%)	15.66 ± 1.27	34.90 ± 6.74
Interocular refractive difference (D)	−0.066 ± 0.043	−0.123 ± 0.202
Physician-diagnosed cataract (%)	25.86 ± 1.61	42.60 ± 7.23
History of cataract surgery (%)	4.59 ± 0.77	9.62 ± 4.60

Values are presented as survey-weighted means or percentages ± standard errors. Sample sizes (*n*) represent unweighted participant counts. The interocular difference in absolute SER was calculated by subtracting the absolute SER of the AMD-free fellow eye from that of the AMD-affected eye. Information on physician-diagnosed cataract was available for 1023 participants with early AMD and 61 participants with late AMD. AMD, age-related macular degeneration; SER, spherical equivalent refraction; D, diopter.

**Table 2:** Interocular visual acuity differences in unilateral early and late age-related macular degeneration.

Visual Acuity Measure	Early AMD	Late AMD	*p* Value
Participants with quantifiable VA in both eyes (*n*)	1035	48	
Mean interocular difference (logMAR)	−0.004 ± 0.006	0.184 ± 0.055	<0.001
95% CI	−0.016 to 0.008	0.077 to 0.291	
**AMD-affected eye worse than fellow eye, weighted % (95% CI)**			
Participants in the full cohort (*n*)	1049	63	
≥0.1 logMAR	17.12 (14.46–19.77)	61.85 (48.29–75.42)	<0.001
≥0.2 logMAR	10.15 (8.17–12.14)	58.31 (44.32–72.29)	<0.001
≥0.3 logMAR	4.54 (3.18–5.90)	46.28 (31.93–60.63)	<0.001

Values are presented as survey-weighted means ± standard errors or survey-weighted percentages (95% CIs). Sample sizes represent unweighted participant counts. Mean interocular differences were calculated only for participants with quantifiable VA in both eyes. Threshold analyses included the full unilateral AMD cohort, including participants with off-chart VA in either eye. When VA in the AMD-affected eye was off-chart and fellow-eye VA was quantifiable, the affected eye was classified as worse at all three thresholds; the converse pattern was classified as not meeting the directional outcome. The interocular difference was calculated as AMD-affected-eye VA minus fellow-eye VA; positive values indicate worse VA in the AMD-affected eye. *p* values represent comparisons between the early and late AMD groups using design-based Wald tests. AMD, age-related macular degeneration; CI, confidence interval; VA, visual acuity.

**Table 3:** Association between late AMD and worse visual acuity in the AMD-affected eye.

Analysis	*n*	Adjusted PR (95% CI)	*p* Value
**A. Analyses using the ≥0.2 logMAR threshold**			
Model 1	1112	5.31 (3.72–7.60)	<0.001
Model 2	1112	5.02 (3.49–7.21)	<0.001
Model 3	1112	4.95 (3.46–7.09)	<0.001
Model 3 + cataract	1084	5.29 (3.72–7.53)	<0.001
Model 3, excluding other retinal diseases	906	5.20 (3.28–8.23)	<0.001
Model 3 + VA testing condition	1109	5.01 (3.48–7.21)	<0.001
**B. Threshold sensitivity analyses using Model 3**			
≥0.1 logMAR	1112	3.26 (2.42–4.37)	<0.001
≥0.2 logMAR	1112	4.95 (3.46–7.09)	<0.001
≥0.3 logMAR	1112	7.77 (4.78–12.63)	<0.001
**C. Threshold sensitivity analyses using Model 3 among participants with quantifiable VA in both eyes**			
≥0.1 logMAR	1083	2.93 (2.06–4.17)	<0.001
≥0.2 logMAR	1083	4.49 (2.94–6.87)	<0.001
≥0.3 logMAR	1083	6.29 (3.42–11.56)	<0.001

Early AMD was used as the reference group. The outcome was defined as VA worse in the AMD-affected eye than in the fellow eye by the specified logMAR threshold or as off-chart VA in the AMD-affected eye when VA in the fellow eye was quantifiable. Model 1 was adjusted for age and sex. Model 2 was additionally adjusted for anisometropia. Model 3 was additionally adjusted for the interocular refractive difference. Model 3 plus cataract variables was further adjusted for cataract diagnosis and history of cataract surgery. Threshold sensitivity analyses were performed using Model 3. Threshold sensitivity analyses in Section C were restricted to participants with quantifiable VA in both eyes. In another sensitivity analysis using Model 3, participants with retinal diseases other than AMD were excluded. The VA-testing-condition sensitivity analysis excluded participants with different or unavailable VA testing conditions between eyes and additionally adjusted for habitual optical correction during VA testing. Sample sizes represent unweighted participant counts. AMD, age-related macular degeneration; CI, confidence interval; PR, prevalence ratio; VA, visual acuity.

**Table 4:** Interocular differences in refractive status among participants with unilateral age-related macular degeneration.

Classification	Early AMD Difference (95% CI)	Late AMD Difference (95% CI)
**Spherical equivalent refraction**		
Myopia (≤−0.50 D)	1.96 (−1.02 to 4.95)	0.52 (−14.85 to 15.89)
Emmetropia (−0.50 < SER < +0.50 D)	−1.45 (−4.95 to 2.04)	−2.60 (−18.61 to 13.40)
Hyperopia (≥+0.50 D)	−0.51 (−2.94 to 1.93)	2.08 (−8.36 to 12.53)
**Astigmatism**		
<0.50 D	0.79 (−1.68 to 3.26)	0.69 (−8.95 to 10.33)
≥0.50 to <1.00 D	−0.67 (−4.83 to 3.49)	−8.08 (−25.52 to 9.36)
≥1.00 to <2.00 D	0.30 (−4.02 to 4.62)	7.17 (−10.77 to 25.11)
≥2.00 D	−0.42 (−3.20 to 2.35)	0.22 (−11.28 to 11.72)

Values are complex survey-weighted paired percentage-point differences (95% CIs), calculated as the prevalence in the AMD-affected eye minus that in the AMD-free fellow eye. Refractive status was classified using SER, and astigmatism severity was classified using absolute cylinder power after standardization to minus-cylinder notation. These refractive-status analyses were exploratory, and no adjustment for multiple comparisons was applied. Because the estimates for the late AMD group had wide confidence intervals, nonsignificant findings should not be interpreted as evidence of equivalence or as excluding clinically relevant interocular differences. AMD, age-related macular degeneration; CI, confidence interval; D, diopter; SER, spherical equivalent refraction.

## Data Availability

The KNHANES data analyzed in this study are publicly available from the Korea Disease Control and Prevention Agency through the KNHANES website (https://knhanes.kdca.go.kr/knhanes/ (accessed on 25 September 2026)) upon completion of the required data-use procedures.

## Supplementary Materials

The following supporting information can be downloaded at: https://www.mdpi.com/article/10.3390/jcm15197672/s1, Table S1: Comparison of participants included in the complete-case analysis and those excluded because of missing data; Table S2: Survey-weight diagnostics and exploratory subtype-specific analyses of late age-related macular degeneration; Table S3: Sensitivity analyses of refractive-covariate specification and survey-year adjustment for the association between late AMD and worse visual acuity.

## Abbreviations

The following abbreviations are used in this manuscript:

AMD	Age-related macular degeneration
CI	Confidence interval
D	Diopter
KNHANES	Korea National Health and Nutrition Examination Survey
logMAR	Logarithm of the minimum angle of resolution
OCT	Optical coherence tomography
PR	Prevalence ratio
RPE	Retinal pigment epithelium
SER	Spherical equivalent refraction
VA	Visual acuity
