# Supplementary material for: Interocular Differences in Visual Acuity and Refractive Status Between Unilateral Early and Late Age-Related Macular Degeneration: A Nationwide Population-Based Study
Source: J Clin Med. 2026 Oct 3;15(19):7672. doi: 10.3390/jcm15197672 (PMC13665769; doi:10.3390/jcm15197672)
Supplement: Supplementary file 1 [file jcm-15-07672-s001.zip › jcm-4583175-supplementary.pdf]

Supplementary Materials

Table S1. Comparison of participants included in the complete-case analysis and those excluded because of missing data

**Characteristic**	Complete-case participants	Participants excluded because of missing data
Participants (n)	11,990	4,164
Age, survey-weighted mean (years)	57.89	56.52
Female, survey-weighted (%)	52.51	49.93
Participants with ascertainable AMD status (n)	11,990	3,102
Any AMD among participants with ascertainable AMD status, survey-weighted (%)	15.24	11.29
**Variables contributing to exclusion, n**		
AMD status unavailable	-	1,062
Bilateral VA data unavailable	-	192
Bilateral refraction data unavailable	-	3,436
Age or sex data unavailable	-	0

Complete-case participants had valid bilateral AMD grading, bilateral visual acuity measurements, bilateral refraction measurements, age, and sex data. Participants excluded because of missing data lacked at least one of these required variables. Sample sizes represent unweighted participant counts, whereas age and percentages are survey-weighted estimates. The prevalence of any AMD was calculated only among participants with ascertainable AMD status. Any AMD was defined as early or late AMD in either eye. This comparison was descriptive, and no formal statistical testing was performed. Missing-data categories were not mutually exclusive because some participants had missing data for more than one variable. Unavailable bilateral VA or refraction data indicate that the required measurement was missing in one or both eyes.

AMD, age-related macular degeneration; VA, visual acuity.

Table S2. Survey-weight diagnostics and exploratory subtype-specific analyses of late age-related macular degeneration

A. Survey-weight diagnostics

AMD group	Unweighted n	Kish effective sample size	ESS/unweighted n, %
Early AMD	1,049	758.55	72.31
Neovascular AMD	43	30.62	71.20
Geographic atrophy	20	16.26	81.32
Late AMD, combined	63	46.51	73.83

B. Subtype-specific descriptive visual-acuity outcomes

Late-AMD subtype	Total n	Mean interocular difference, logMAR (95% CI)	≥0.2 logMAR worse in AMD-affected eye, weighted % (95% CI)	Off-chart VA in AMD-affected eye, n
Neovascular AMD	43	0.188 (0.047-0.330)	57.04 (39.37-74.70)	9
Geographic atrophy	20	0.175 (0.028-0.323)	60.30 (36.20-84.40)	6

C. Exploratory subtype-specific associations with worse visual acuity in the AMD-affected eye

Comparison	n	Adjusted PR (95% CI)	P value
Neovascular AMD vs. early AMD	1,092	4.84 (3.21-7.29)	<0.001
Geographic atrophy vs. early AMD	1,069	5.13 (3.12-8.44)	<0.001

Effective sample sizes were estimated using the Kish approximation based on the survey weights. Sample sizes represent unweighted participant counts. Mean interocular differences are presented as survey-weighted means with 95% confidence intervals and were calculated only for participants with quantifiable VA in both eyes (34 with neovascular AMD and 14 with geographic atrophy). The proportions meeting the ≥0.2 logMAR threshold are presented as survey-weighted percentages with 95% confidence intervals and included participants with off-chart VA in the AMD-affected eye when fellow-eye VA was quantifiable. Off-chart counts represent unweighted participant counts. Early AMD was used as the reference group in the subtype-specific regression analyses. The outcome was defined as VA in the AMD-affected eye that was ≥0.2 logMAR worse than that in the fellow eye or as off-chart VA in the AMD-affected eye when fellow-eye VA was quantifiable. Prevalence ratios were estimated using survey-weighted modified Poisson regression and adjusted for age, sex, anisometropia, and the interocular refractive difference. Subtype-specific analyses were exploratory because of the small numbers of participants with neovascular AMD and geographic atrophy.

AMD, age-related macular degeneration; CI, confidence interval; ESS, effective sample size; logMAR, logarithm of the minimum angle of resolution; PR, prevalence ratio; VA, visual acuity.

Table S3. Sensitivity analyses of refractive-covariate specification and survey-year adjustment for the association between late AMD and worse visual acuity

Analysis	n	Adjusted PR (95% CI)	P value
**A. Refractive-covariate specification**			
Anisometropia and interocular difference in absolute SER	1,112	4.95 (3.46-7.09)	<0.001
Continuous anisometropia and interocular difference in absolute SER	1,112	5.37 (3.76-7.67)	<0.001
Anisometropia only	1,112	5.02 (3.49-7.21)	<0.001
Interocular difference in absolute SER only	1,112	5.21 (3.66-7.42)	<0.001
Neither refractive covariate	1,112	5.31 (3.72-7.60)	<0.001
**B. Additional adjustment for survey year**			
Model 3	1,112	4.95 (3.46-7.09)	<0.001
Model 3 + survey year	1,112	5.42 (3.71-7.91)	<0.001

Early AMD was used as the reference group. The outcome was defined as visual acuity in the AMD-affected eye that was ≥0.2 logMAR worse than that in the fellow eye or as off-chart visual acuity in the AMD-affected eye when fellow-eye visual acuity was quantifiable. Prevalence ratios were estimated using survey-weighted modified Poisson regression. All models were adjusted for age and sex. The models in Section A differed according to the inclusion and specification of the refractive covariates. Anisometropia was defined as an absolute interocular SER difference of ≥1.0 D. Continuous anisometropia was defined as the absolute interocular difference in SER. The interocular difference in absolute SER was calculated as the absolute SER of the AMD-affected eye minus that of the fellow eye. Model 3 included age, sex, anisometropia, and the interocular difference in absolute SER. Survey year was additionally included as a categorical variable in the final sensitivity model. Sample sizes represent unweighted participant counts.

AMD, age-related macular degeneration; CI, confidence interval; logMAR, logarithm of the minimum angle of resolution; PR, prevalence ratio; SER, spherical equivalent refraction; VA, visual acuity.
